# Using of geographic information system for risk area analysis of liver flukes in Thailand

**DOI:** 10.1186/2047-2994-4-S1-P116

**Published:** 2015-06-16

**Authors:** N Kaewpitoon, S Kaewpitoon, R Rujirakul

**Affiliations:** 1 Public Health, Vongchavalitkul University, Nakhon Ratchasima, Thailand; 2 Family Medicine and Community Medicine, Nakhon Ratchasima, Thailand; 3 Parasitic Infectious Disease Research Unit, Institute of Medicine, Nakhon Ratchasima, Thailand

## Introduction

*Opisthorchis viverrini* is associated with cholangiocarcima and its high incidence in Thailand.

## Objectives

This study aims to investigate the human behavior, and environmental factors influencing to the distribution, and to build a model using stepwise multiple regression analysis with geographic information systems on environment and climate data.

## Methods

GIS was used for analysed the risk areas in Surin province of Thailand, from 2012 and 2013 including: human behaviors (knowledge, attitudes, and practice). Liver fluke infections were screened from 40 cases/districts) by Kato’s thick smear. The relationship between liver fluke and human behavior, health service unit, and environmental factors using statistic analysis by stepwise multiple correlation.

## Results

The *O. viverrini* infection was found in 46 from 680 eligible participants. The human behavior; attitudes, was correlated with the liver fluke disease distribution at 0.000 level, while, the site of health service unit were not correlated with the liver fluke disease distribution. The relationship between the environmental factors; population density (148-169 pop/km^2^; X_73_), was correlated with the liver fluke disease distribution at 0.034 level. Land use factor has significantly correlation between wetland (X_64_), and liver fluke disease distribution at 0.006 level. The multiple regression analysis method was used to predict the distribution of liver fluke. Equation following: OV = -.599 + 0.005(population density (148-169 pop/km^2^); X73) + 0.040(human attitude (<50%; X_111_) +0.022(land used (wetland; X_64_), OV is the patients of liver fluke infection, R Square= 0.878, and, Adjust R Square= 0.849. By equation, it was found population density (148-169 pop/km^2^), human attitude <50%, land used; wetland were effect on the disease dispersion.

## Conclusion

Combination of GIS and statistical analysis which helps to simulate the spatial distribution and risk areas of liver fluke, is a potential tool for future planning a prevention and control.

## Disclosure of interest

None declared.
